# Hippocampal atrophy is associated with psychotic symptom severity following traumatic brain injury

**DOI:** 10.1093/braincomms/fcab026

**Published:** 2021-03-09

**Authors:** Michael J C Bray, Bhanu Sharma, Julia Cottrelle's, Matthew E Peters, Mark Bayley, Robin E A Green

**Affiliations:** 1 Institute of Medical Science, University of Toronto, Toronto, ON M5S 1A8, Canada; 2 Toronto Rehabilitation Institute, University Health Network, Toronto, ON M5G 2A2, Canada; 3 Department of Psychiatry and Behavioral Sciences, Johns Hopkins University School of Medicine, Baltimore, MD 21224, USA; 4 The KITE Research Institute—University Health Network, Toronto, ON M5G 2A2, Canada; 5 Department of Medical Sciences, McMaster University, Hamilton, ON L8S 4L8, Canada

**Keywords:** hippocampus, aberrant salience, psychosis, traumatic brain injury, neurodegeneration

## Abstract

Psychosis is a rare, but particularly serious sequela of traumatic brain injury. However, little is known as to the neurobiological processes that may contribute to its onset. Early evidence suggests that psychotic symptom development after traumatic brain injury may co-occur with hippocampal degeneration, invoking the possibility of a relationship. Particularly regarding the hippocampal head, these degenerative changes may lead to dysregulation in dopaminergic circuits, as is reported in psychoses due to schizophrenia, resulting in the positive symptom profile typically seen in post-injury psychosis. The objective of this study was to examine change in hippocampal volume and psychotic symptoms across time in a sample of moderate-to-severe traumatic brain injury patients. We hypothesized that hippocampal volume loss would be associated with increased psychotic symptom severity. From a database of *n* = 137 adult patients with prospectively collected, longitudinal imaging and neuropsychiatric outcomes, *n* = 24 had complete data at time points of interest (5 and 12 months post-traumatic brain injury) and showed increasing psychotic symptom severity on the Personality Assessment Inventory psychotic experiences subscale of the schizophrenia clinical scale across time. Secondary analysis employing stepwise regression with hippocampal volume change (independent variable) and Personality Assessment Inventory psychotic symptom change (dependent variable) from 5 to 12 months post-injury was conducted including age, sex, marijuana use, family history of schizophrenia, years of education and injury severity as control variables. Total right hippocampal volume loss predicted an increase in the Personality Assessment Inventory psychotic experiences subscale (*F*_(1, 22)_ = 5.396, adjusted *R*^2^ = 0.161, *P *=* *0.030; *β* = −0.017, 95% confidence interval = −0.018, −0.016) as did volume of the right hippocampal head (*F*_(1, 22)_ = 5.764, adjusted *R*^2^ = 0.172, *P *=* *0.025; *β* = −0.019, 95% confidence interval = −0.021, −0.017). Final model goodness-of-fit was confirmed using *k*-fold (*k* = 5) cross-validation. Consistent with our hypotheses, the current findings suggest that hippocampal degeneration in the chronic stages of moderate-to-severe traumatic brain injury may play a role in the delayed onset of psychotic symptoms after traumatic brain injury. These findings localized to the right hippocampal head are supportive of a proposed aetiological mechanism whereby atrophy of the hippocampal head may lead to the dysregulation of dopaminergic networks following traumatic brain injury; possibly accounting for observed clinical features of psychotic disorder after traumatic brain injury (including prolonged latency period to symptom onset and predominance of positive symptoms). If further validated, these findings may bear important clinical implications for neurorehabilitative therapies following traumatic brain injury.

## Teaser

Bray et al. report psychotic symptom severity was associated with volumetric loss in the right hippocampal head across the chronic stages of moderate–severe traumatic brain injury. The authors propose a novel aetiological hypothesis by which this localized neurodegenerative change promotes dopaminergic network dysregulation contributing to delayed positive symptomatology post-brain injury.

## Graphical Abstract

**Graphical Abstract:**
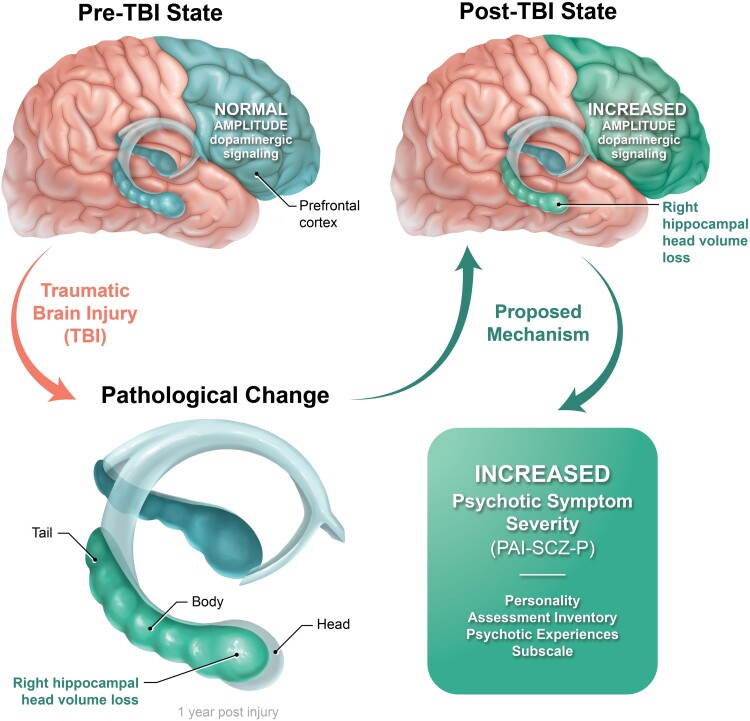

## Introduction

Schizophrenia-like psychosis, largely comprising hallucinations and delusions,^1–3^ is a rare, but exceptionally debilitating sequela of traumatic brain injury (TBI), eroding the quality of life and activities of daily living.^4^^,^^5^ Following TBI, risk of psychosis is increased significantly, with one meta-analysis finding the odds of incidence to increase by 65%.^6^ The cause of psychosis after TBI is currently unclear, including whether underlying, progressive, organic changes secondary to TBI precipitate this increased risk. Here, we focus on the hippocampi, given their known role in the neuropathophysiology of the positive symptoms of schizophrenia and observed atrophy of these structures along similar timelines as has been reported for onset of post-TBI psychosis.

Interestingly, onset of psychosis following TBI generally occurs after a latency period of 1–3 years post-injury.^7–11^ This latency period suggests that the aetiology of psychosis following TBI may not depend on primary injuries, but rather on post-acute changes, as risk continues to increase even as resolution of primary injuries is occurring.^7^^,^^8^^,^^10^^,^^12^ In the context of schizophrenia without TBI history, onset of psychotic symptoms is widely understood to involve neurodevelopmental timelines, with genetic polymorphisms contributing to altered brain development.^13^^,^^14^ Consequently, the majority of these cases present between 16 and 25 years of age.^15^ In TBI, however, there is a great degree of heterogeneity in age at onset, which may often appear dependent on age of injury^16^ and is commonly reported in older individuals.^11^ The current evidence surrounding time at onset of psychosis following TBI is highly heterogenous, making definitive epidemiological estimates challenging.

One possible explanation for this delayed onset is ongoing degenerative change in the brain (and as discussed below, the hippocampi). While TBI is known to alter neuropsychiatric symptomatology in the context of neurodegenerative conditions,^17^ TBI itself is becoming increasingly understood as not simply an acute injury, but rather an ongoing neurodegenerative process.^18^^,^^19^ There is a burgeoning literature showing deteriorative changes in the brain during the chronic stages of moderate–severe TBI,^20–23^ with one study finding 96% of patients demonstrating atrophic change between 5 and 20 months post-injury.^21^ Such degenerative changes include long-standing neuroinflammatory processes,^24–26^ both grey and white matter atrophy,^27^ whole brain atrophy, including measures of ventricle-to-brain ratio^28^ and discrete structures of the brain, including the hippocampus, corpus callosum and fornix.^20^^,^^21^

As in schizophrenia, psychosis after TBI is commonly preceded by prodromal symptoms.^8^ Predominantly positive symptoms (hallucinations and delusions) are observed, with auditory hallucinations as well as paranoid and persecutory delusions reported as the most common features.^1–3^ These symptoms of psychosis following TBI are observed to respond comparably to D2 receptor antagonism by either typical or atypical anti-psychotics,^29^^,^^30^ suggesting a pathophysiological role for dopaminergic system dysregulation, and specifically increased amplitude of mesolimbic dopaminergic firing in response to inappropriate stimuli, as is understood to underlie positive symptoms of schizophrenia.^31–33^

Distinct from schizophrenia, however, multiple studies have found a scarcity of negative symptoms (e.g. apathy and withdrawal) accompanying post-TBI psychosis.^1^^,^^8^^,^^34^ While dopaminergic dysregulation is regarded as a driving force underlying positive symptoms,^31–33^ negative symptoms are associated with differing neurochemical dysfunction, with less predominance of dopaminergic systems, and including important contributions from serotonergic, glutamatergic and cholinergic systems.^35–38^ The relative rarity of negative symptoms in individuals experiencing post-TBI psychosis may therefore speak to an underlying pathophysiology preferentially influencing dopaminergic networks.

Perhaps similar to psychosis following TBI, neurodevelopmental timelines are not responsible for psychosis onset in neurodegenerative conditions such as Alzheimer’s and Parkinson’s diseases. Rather, psychotic symptoms develop due to neurodegenerative changes.^39^^,^^40^ Emerging evidence suggests that degenerative change within the hippocampal formation is particularly associated with psychoses in dementia.^41^^,^^42^ Perhaps relatedly, the timelines of volumetric decline in the hippocampal formation (in the chronic phase of TBI), occur within the same time period as increasing risk of psychosis post-TBI.^6^^,^^21^^,^^28^^,^^34^^,^^43^ These findings converge with the possibility that hippocampal volume loss contributes to psychosis after TBI.

The hippocampi play an important role in the mediation of affective salience (the motivational importance of a given stimulus) through the control of dopaminergic firing to the prefrontal cortex.^44^ Notably, dysfunction in assignment of affective salience is understood to be a key contributor to the precipitation of hallucinations and delusions.^31–33^ Inhibitory control neurons, important to the regulation of this neurochemical system, are arranged in a longitudinal gradient, being predominately localized to the hippocampal head,^45–47^ with a declining concentration of these neurons also seen in the hippocampal body and a relative scarcity within the tail.^48^ Degenerative change in these key regulatory neurons has been linked to elevated psychotic symptom severity in the context of schizophrenia^44^^,^^47^^,^^49^ and thus warrants consideration in the context of TBI-related degenerative change.

While post-TBI psychosis has been observed following both left- and right-sided damage to the brain, Fujii and Ahmed^34^ found right-sided focal damage to be more commonly associated with psychotic disorder after TBI. This is consistent with the lateralization observed following other acquired brain injuries such as stroke, in which right-sided (as opposed to left-sided) damage has been repeatedly associated with schizophreniform psychosis.^50–52^ Finally, studies in Alzheimer’s disease have reported right-sided hippocampal atrophy to be associated with psychosis.^42^

Taken together, literature demonstrates that hippocampal atrophy may occur in the chronic stages of moderate-to-severe TBI, that dysfunction of the hippocampal head plays a role in the pathophysiology of positive psychotic symptoms and that psychosis following TBI occur along the timelines of hippocampal atrophy with predominantly positive psychotic symptoms (particularly in right-sided brain injury). Collectively, this highlights a potential role for right hippocampal degenerative change in the elevation of post-TBI psychosis risk.

To explore this possibility, we undertook a secondary analysis of a prospectively collected, longitudinal data set of participants with history of moderate-to-severe TBI for whom quantitative MRI of the hippocampi and a validated measure of psychotic symptom severity [Personality Assessment Inventory (PAI)]^53^ were available at two timepoints. Hippocampal volumetric change from 5 to 12 months post-injury was compared to change on the Psychotic Experience Subscale on the Schizophrenia Clinical Scale of the PAI (PAI-SCZ-P) measured at the same timepoints. We predicted that right-sided hippocampal volume loss (and specifically in the hippocampal head) would be associated with worsening (i.e. increasing) scores on the PAI-SCZ-P across the two time points.

A hypothesis may be further supported by establishing divergent validity (also known as discriminant validity), a construct by which outcomes hypothesized to be unrelated to one another are found to have no significant association. Given our primary hypothesis (namely, that a specific pattern of atrophy within the right hippocampal head is related to psychotic symptom severity), we further hypothesized that: (1) psychotic symptom severity would be unrelated to degenerative change in the corpus callosum, a structure which declines along similar timelines as the hippocampus but which is unrelated to the proposed mechanism of post-TBI psychotic symptomatology and (2) patterns of hippocampal atrophy associated with psychotic symptom severity would be unrelated to negative symptomatology (as measured by the Social Detachment Subscale of the Schizophrenia Clinical Scale of the PAI [PAI-SCZ-S]), which is notably uncommon in psychoses following TBI. Through these secondary aims we sought to provide evidence against the potential alternative explanations that (1) psychotic symptom severity following TBI is associated with severity of brain atrophy in general and not patterns of hippocampal atrophy specifically and (2) atrophy of the right hippocampal head associated with worsening behavioural outcomes broadly as opposed to specific patterns consistent with psychosis following TBI.

## Materials and methods

### Participants

This secondary analysis included participants from a larger, prospective, longitudinal cohort study^54^^,^^55^ of *n* = 137 individuals with clinically confirmed, moderate-to-severe TBI (see inclusion criteria). Participants in this study were recruited from the inpatient acquired brain injury neurorehabilitation programme of a large, urban hospital in Toronto, Canada. Informed consent was obtained for all participants. Participants in the study underwent neuropsychological assessment, neuropsychiatric assessment (including the PAI), and neuroimaging (including quantitative MRI) at multiple timepoints, including 5- and 12-months post-injury. Inclusion criteria for the parent cohort comprised: (1) acute care diagnosis of moderate-to-severe TBI; (2) post-traumatic amnesia (PTA) ≥24 h and/or lowest Glasgow Coma Score <13; and (3) age ≥18 years. An exclusion criterion of the parent study was history of pre-TBI psychotic disorder. See Green^54^ and Graves and Green^55^ for full list of parent study inclusion and exclusion criteria. Inclusion criteria of the present study were availability of MRI data and PAI scores at 5 and 12 months post-TBI. An exclusion criterion of the present study was improvement (as opposed to stability or decline) on the PAI-SCZ-P, the primary outcome of the current study. By the hypothesized relationship, hippocampal atrophy is related to decline on the PAI-SCZ-P but is unrelated to improvement. As such, participants with improvement on the PAI-SCZ-P were not relevant to primary hypothesis testing and were excluded. Injury and demographic data were collected from clinical interview and medical chart review (Table 1).

Of 137 participants included in the parent study, 87 were excluded for incomplete data and 25 were excluded as improvers on the PAI-SCZ-P between 5 and 12 months. A total of 25 participants met all inclusion and exclusion criteria and were eligible for the current study.

**Table 1:** Participant characteristics

	Mean (SD)	Range
Demographics		
Age	37.3 (16.0)	18, 68
% female	25.0	–
Years of education	14.5 (2.55)	9, 18
Injury severity		
GCS score	7.89 (3.73)	3, 13
PTA duration (%)		
1–7 days	46.7%	–
1–4 weeks	26.1%	–
>4 weeks	26.1%	–
PAI-SCZ-P T-scores		
5 months	41.8 (4.76)	36, 50
12 months	47.4 (8.58)	36, 73
% change	13.3 (13.7)	0, 46
Right HPC % change (volume) 5–12 months		
Head	−2.63 (3.32)	−13.0, 1.69
Body	−4.06 (4.71)	−15.9, 0.65
Tail	−5.04 (5.81)	−18.6, 0.64
Total	−3.37 (3.61)	−14.9, 0.78
Left HPC % change (volume) 5–12 months		
Head	−2.38 (1.87)	−6.19, 0.18
Body	−3.02 (3.39)	−13.7, 1.63
Tail	−4.70 (5.55)	−20.2, 0.96
Total	−2.73 (2.21)	−8.61, 0.02

Participant demographics (*N* = 24) and outcome measure characterization. GCS = Glasgow Coma Score; HPC, hippocampus.

### Outcome measures

#### Primary predictor: neuroimaging of hippocampi

The primary predictor variable of interest was volumetric change of the hippocampus and substructures between 5 and 12 months post-TBI. Details of the quantitative MRI of the hippocampi have been described in in full previously.^21^ In brief, MRI scans were obtained using a General Electric Signa-Echospeed 1.5 Tesla HD scanner (eight-channel head coil). Sequences comprised sagittal T_1_, Repetition Time (TR)/Echo Time (TE) = 300/13 ms, slice thickness = 5 mm, space 2.5 mm, matrix 256 × 128, axial gradient recalled-echo, TR/TE = 450/20 ms, flip angle = 20°, slice thickness = 3 mm (no gap), matrix 256 × 192, axial fluid-attenuated-inversion-recovery, TR/TE = 9000/45 ms, inversion time = 2200 ms, slice thickness = 5 mm (no gap), matrix 256 × 192, axial fast spin echo proton density/T_2_, TR/TE = 5500/30/90 ms, slice thickness = 3 mm (no gap), matrix 256 × 192. A 22-cm field of view was used to obtain above sequences. A 25-cm field of view was used to acquire high-resolution, axial, isotropic T_1_-weighted, three-dimensional IR prepped radio-frequency spoiled-gradient recalled-echo (3D IRSPGR) images with inversion time/TR/TE = 12/300/5, FA = 20, slice thickness = 1 mm (no gap), matrix = 256 × 256. Imaging processing is described in Green et al.^21^ and was performed in accordance with guidelines of Pruessner et al.^56^ An experienced tracer performed voxel-wise, manual segmentation of the hippocampus and corpus callosum using Analyze TM 8.1 (Brain Imaging Resource, Mayo Clinic, MN) in accordance with protocols described by Watson et al.^57^ and Maller et al.^58^ The hippocampal head and body were measured in coronal section from the anterior tip of the hippocampus to the slice immediately anterior to the crux of the fornix.^58^ The tail was measured from the opening of the crux of the fornix to the last slice of the hippocampus according to the Watson protocol.^57^^,^^58^

#### Primary response: percent change in psychosis over time

The PAI assesses psychotic experiences and social detachment using eight non-overlapping items each, which are rated by participants on a 4-point Likert scale. These items include statements such as ‘I’ve heard voices no one else can hear’ (PAI-SCZ-P) and ‘I don’t feel close to anyone’ (PAI-SCZ-S). The PAI is a self-administered assessment with demonstrated validity and reliability for use in a general population of adults aged 18 years or older, as well as in a sample of psychiatric inpatients (including patients experiencing schizophrenia), in which full scale and subscale reliabilities of individual clinical scales, including PAI-SCZ-P and PAI-SCZ-S, were reported to be large and acceptable.^53^^,^^59^ PAI-SCZ-P and PAI-SCZ-S subscales include no transdiagnostic items for TBI and have been validated for use in moderate-to-severe TBI. Though several items which make up the larger Schizophrenia Clinical Scale have been found to be transdiagnostic for TBI,^60^ the affected subscale was not used in these analyses and no correction was required. T-scores are calculated for the PAI-SCZ-P and PAI-SCZ-S using normative data provided in Morey 2014 for clinical scales and subscales. As such, these subscales represent continuous measures of symptom severity and may not be used for diagnosis. This continuous approach to evaluation of psychotic symptomatology, as opposed to a dichotomous one, permits the evaluation of intra-participant change over time. Change scores from 5 to 12 months post-injury (computation below) using T-scores for the PAI-SCZ-P and the PAI-SCZ-S were the response variables used in the present analyses. The PAI-SCZ-P is designed to measure positive symptoms of schizophrenia including unusual perceptions, magical thinking, and frank delusions or hallucinations. Items are relatively specific to the positive symptoms of schizophrenia and have minimal overlap with, for instance, delusions which might be found in other psychiatric syndromes (e.g. delusions of grandeur). The primary response variable of interest was percent change in the PAI-SCZ-P^53^ between 5 and 12 months post-TBI.

### Control variables

This investigation also accounted for injury severity as well as variables known to influence risk of psychosis and/or neurodegeneration. As such, injury severity, assessed by duration of PTA, as well as age, years of education, family history of psychotic disorder and history of pre-injury marijuana use were included for analysis as control variables.^61^^,^^62^ Duration of PTA (documented during inpatient stay) was obtained by medical chart review. Length of PTA is a superior predictor of clinical outcomes as compared to other metrics of injury severity, such as the Glasgow Coma Score.^63–66^ Family history of psychotic disorder was assessed as a dichotomous variable obtained by clinical interview and medical chart review. Pre-injury marijuana use was assessed using T-scores on the Drug Problems Scale of the PAI^53^ in conjunction with clinical interview to verify type of drugs used. In this sample, only marijuana use was reported. These control variables were included in all regression analyses performed in the present investigation.

### Divergent validity outcomes

Negative symptomatology was assessed using the Social Detachment Subscale of the Schizophrenia Clinical Scale (PAI-SCZ-S). The PAI-SCZ-S is designed to measure the most characteristic negative symptoms of schizophrenia including social withdrawal, poor rapport and affective responsivity.^53^

Volumetric change of the corpus callosum between 5 and 12 months post-TBI was included as an additional predictor variable in analyses including right and left total hippocampal volume as primary predictors. As with hippocampus, details of the quantitative MRI (1.5 T) of the corpus callosum and image processing have been described in detail previously.^21^ Manual segmentation of the corpus callosum was performed by an experienced tracer in sagittal section using the following anatomical landmarks: no white matter (or minimal white matter in the cortical mantle surround the corpus callosum), interthalamic adhesion, transparent septum and visibility of the cerebral aqueduct.

### Data preparation

Change scores from 5 to 12 months post-injury were computed on the T-scores of the PAI-SCZ-P and PAI-SCZ-S subscales. The change score was computed using the following formula: (T_2_ − T_1_)/T_1_ × 100^67^; where T_1_ represents the PAI value at 5 months post-injury and T_2_ represents the PAI value at 12 months post-injury. Changes scores for the hippocampi total scores and substructures and for the total corpus callosum between 5- and 12-month time-points were also computed. The change score was computed using the following formula: (T_2_ − T_1_)/[(T_1_ + T_2_)/2] × 100; where T_1_ represents value at 5 months post-injury and T_2_ represents value at 12 months post-injury, as is precedented in the literature.^21^

### Statistical analyses

Secondary analyses were performed on prospectively collected, longitudinal data. Within-subject analyses were performed on the data with primary predictor variables consisting of volumetric change scores of the right and left total hippocampi, the head, body and tail of the hippocampi, and the total corpus callosum. Response variables were change scores on the PAI-SCZ-P and PAI-SCZ-S. Control variables are described above. The corpus callosum was included in stepwise regression analysis including total right and left hippocampus to evaluate whether PAI-SCZ-P scores were associated with specific patterns of degenerative change (for instance, in the hippocampus) as opposed to poor organic outcomes broadly. Notably, the corpus callosum has been observed to undergo degenerative change along the same timelines as the hippocampus and therefore was selected for analysis.^21^

Statistical analyses were performed using R version 3.6.1 with a value of α specified *a priori* as 0.05. Hypothesized associations were analysed using bidirectional stepwise regression, minimizing the Bayesian information criterion (BIC) which measures model goodness-of-fit while penalizing the model for over-fitting, thereby resulting in parsimonious models.^68^ Lower BIC scores indicate superior goodness-of-fit and lower tendency of over-fitting data to the model. Given our moderate-sized sample of moderate-to-severe TBI patients (albeit representative of the larger pool of participants from which they are derived, described below), a stepwise selection method that favoured parsimonious models was preferred, as to not underpower the model. First, regression analysis was performed, with volumetric percent change in the corpus callosum (as a measure of divergent validity), total right, and total left hippocampi fed to the stepwise selection process as possible predictor variables, with the PAI-SCZ-P percent change as the response variable. Subsequent stepwise regression of sub-structures was planned based on the outcome of the first regression analysis (i.e. all right, all left or both) and performed.

Resultant models were further assessed for over-fitting using *k*-fold cross-validation (*k* = 5), and performance of cross-validation models was evaluated using methods described by Neumann et al. and Vogel et al.^69^^,^^70^ First, predicted values generated by cross-validation models were assessed for concordance with observed data values using Pearson correlation coefficients. Next, the mean model *R*^2^ across all *k* validation models and corresponding 95% confidence interval (CI) were calculated to obtain a *P*-value. Only a final model whose cross-validation results were assessed to be significant by both tests was considered valid.

As a further index of divergent validity, a stepwise regression was performed including, as the predictor variable(s) the hippocampal substructure(s) associated with PAI-SCZ-P in the prior analysis, as the response variable, PAI-SCZ-S change scores, along with control variables, as in analyses above. This was performed to examine whether patterns of degeneration observed to be associated with PAI-SCZ-P scores were associated with a behavioural presentation typical of post-TBI psychosis or rather poor psychological outcomes broadly.

### Data availability

Deidentified data may be made available upon reasonable request.

## Results

### Study population and characterization

One participant was identified as an outlier on the basis of PAI-SCZ-P scores at 5 and 12 months post-injury, which were >3 z-scores from the mean, as well as volumetric change scores of the right hippocampal head and tail that were >3 and 4 z-scores from the mean, respectively. In preliminary analyses, inclusion of this participant increased effect sizes and decreased *P*-values in favour of the primary hypothesis and so, conservatively, was excluded list-wise from all analyses for a final included cohort of *n* = 24 participants.

Per comparison to patients who were excluded from this study (*n* = 113), there were no significant differences in age, education, gender, or injury severity (*P *>* *0.200 for all comparisons). Participant demographics for the 24 moderate-to-severe TBI patients included in our analysis are summarized in Table 1.

Furthermore, Table 1 summarizes baseline, follow-up, and percent-change values for outcomes of primary interest (namely, PAI-SCZ-P) as well as candidate predictors (left and right segmented hippocampal volume percent change). Volume changes did not differ between in the right hippocampus as compared to the left hippocampus for either total volume or substructures (head, body, or tail). While mean volume changes were negative (i.e. volume loss), there were patients whose hippocampal volumes increased from 5 to 12 months post-injury. This heterogeneity is consistent with patterns of recovery in moderate-to-severe TBI.^27^

### Stepwise regression modelling

Stepwise selection examining volume change in the corpus callosum, as well as in the total right and left hippocampus volume as predictor variables, including BIC scores associated with the addition of each candidate parameter, is presented in Table 2. In step 1, only right hippocampal % volume change is added to the model. In step 2, no further addition of candidate parameters led to a reduction in the BIC. The two-step stepwise approach therefore results in a univariate model that predicts PAI-SCZ-P percent change (from 5 to 12 months post-injury) based on right hippocampal percent volume change across the same time interval (*F*_(1, 22)_* *=* *5.396, adjusted *R*^2^* *=* *0.161, *P *=* *0.030). The parameter estimate for right hippocampal percent volume change was −0.017 (95% CI = −0.031, −0.003, *P *=* *0.029). In a supplemental analysis, this predictor was also found to remain significant in a multivariate model with all control variables forced in (Supplementary Table 1). An additional supplemental stepwise regression analysis found that the corpus callosum did not significantly improve the stepwise model when included in the absence of total right and left hippocampal volume.

**Table 2:** Summary of brain volumetric stepwise linear regression

	Candidate parameter	SS	RSS	BIC
Step 1	Right HPC % volume change, 5–12 months	0.096	0.298	−62.373
	No further addition	–	0.394	−60.814
	Age	0.061	0.333	−60.487
	Left HPC % volume change, 5–12 months	0.026	0.369	−58.774
	Pre-injury marijuana use	0.024	0.370	−58.696
	Sex	0.016	0.378	−58.338
	Education	0.011	0.383	−58.140
	CC % volume change, 5–12 months	0.005	0.389	−57.846
	Family history of Schizophrenia	0.000	0.394	−57.636
	PTA	0.008	0.386	−54.824
Step 2	No further addition	–	0.349	−95.214
	(−) Right HPC % volume change, 5–12 months	0.085	0.434	−93.127
	Pre-injury marijuana use	0.027	0.271	−60.819
	Age	0.022	0.276	−60.499
	Education	0.013	0.286	−59.926
	Left HPC % volume change, 5–12 months	0.010	0.288	−59.926
	Sex	0.009	0.289	−59.739
	Family history of Schizophrenia	0.007	0.292	−59.570
	CC % volume change, 5–12 months	0.001	0.298	−59.224
	PTA	0.033	0.266	−57.980

Stepwise selection method for building a linear regression model to predict PAI-SCZ-P percent change from 5 to 12 months post-injury. CC = Corpus Callosum; HPC = hippocampus; SS = sum of squares; RSS = residual sum of squares. (−) indicates removal from the model of an included variable.

Following the results of the initial regression analysis, a second stepwise regression analysis was performed to examine predictive outcome variance for each of the right hippocampal substructures (i.e. head, body or tail) for PAI-SCZ-P change. Using the same BIC approach (Table 3), we found that PAI-SCZ-P percent change from 5–12 months post-injury was predicted by right hippocampal head percent volume changes across the same interval (*F*_(1, 22)_* *=* *5.764, adjusted *R*^2^* *=* *0.172, *P *=* *0.025). The parameter estimate for right head hippocampal percent volume change was −0.019 (95% CI = −0.035, −0.003, *P *=* *0.025). In a supplemental analysis, this predictor was also found to remain significant in a multivariate model with all control variables forced-in (Supplementary Table 2).

**Table 3:** Summary of segmented hippocampal stepwise linear regression

	Candidate parameter	SS	RSS	BIC
Step 1	Right HPC head % volume change, 5–12 months	0.081	0.335	−90.928
	Age	0.075	0.341	−90.508
	Right HPC body % volume change, 5–12 months	0.060	0.356	−89.532
	No further addition	–	0.416	−89.138
	Sex	0.019	0.396	−87.051
	Right HPC tail % volume change, 5–12 months	0.011	0.405	−86.576
	Pre-injury marijuana use	0.006	0.410	−86.271
	Education	0.003	0.412	−86.145
	Family history of Schizophrenia	0.000	0.415	−85.967
	PTA	0.011	0.405	−83.372
Step 2	No further addition	–	0.344	−95.533
	(−) Right HPC head % volume change, 5–12 months	0.090	0.434	−93.127
	Age	0.042	0.293	−90.796
	Sex	0.031	0.304	−89.976
	Right HPC tail % volume change, 5–12 months	0.016	0.319	−88.865
	Pre-injury marijuana use	0.015	0.320	−88.825
	Right HPC body % volume change, 5–12 months	0.008	0.332	−88.292
	Education	0.007	0.328	−88.217
	Family history of Schizophrenia	0.003	0.332	−87.945
	PTA	0.015	0.320	−85.622

Inclusion of segmented right HPC volume percent change into the stepwise selection method for building a linear regression model to predict PAI-SCZ-P percent change from 5 to 12 months post-injury. HPC = hippocampus; SS = sum of squares; RSS = residual sum of squares. (−) indicates removal from the model of an included variable.

Stepwise regression modelling found that a predictive model of PAI-SCZ-S change scores was not significantly improved by the addition of right hippocampal head percent volume changes (Supplementary Table 3).

### *k*-Fold cross-validation

**Figure 1:**
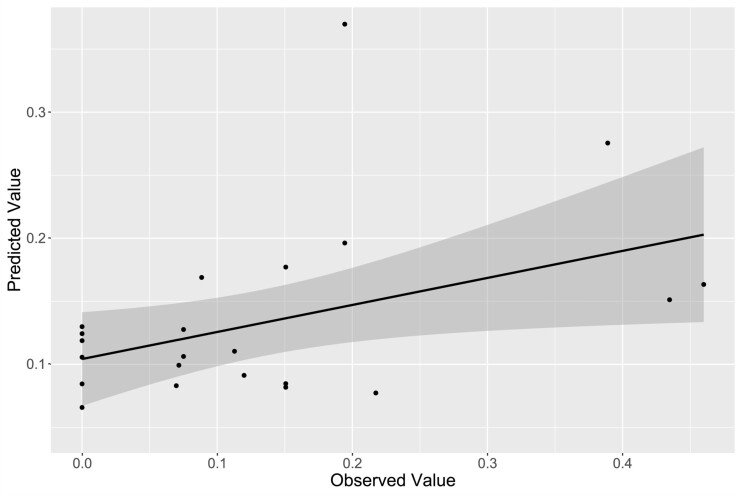
**Correlation of observed and validation model-predicted values.** Comparison of observed PAI-SCZ-P values and predicted values generated by *k*-fold cross-validation of final model produced by the stepwise selection method.

**Figure 2:**
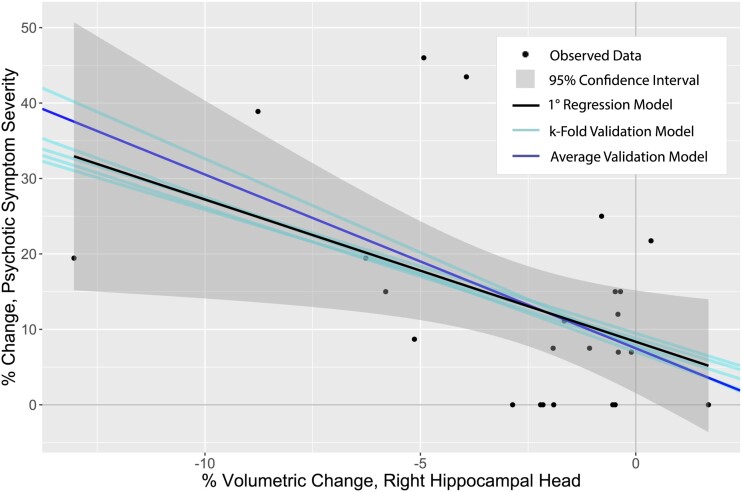
**Regression and validation models.** Comparison of univariate model generated by stepwise selection method and those generated by *k*-fold cross-validation. Percent change in psychotic symptom severity (as measured by PAI-SCZ-P score) on the y-axis is represented by proportions (e.g. a value of 0.5 corresponds to a percent change of 50%).

*k*-Fold cross-validation (*k* = 5) was performed to evaluate goodness-of-fit of the final univariate model identified by stepwise selection (right hippocampal head). Predicted values (generated by cross-validation) demonstrated significant correlation with observed values (*R *=* *0.412, *P *=* *0.046; Fig. 1). Furthermore, *R*^2^ values generated by cross-validation demonstrate acceptability of the model (adjusted *R*^2^* *=* *0.395, 95% CI = 0.152, 0.639, *P *=* *0.001; Fig. 2). Performance of the univariate model (black line, with 95% CI) of psychotic symptom severity as predicted by percent volume change of the right hippocampal head from 5–12 months post-injury can be compared against comparative linear models generated by *k*-fold cross-validation (teal and blue lines; Fig. 2). These results demonstrate similarity between the univariate model based on the observed data, and the models generated by *k*-fold cross-validation (all of which fall within the 95% CI bounds of the observed univariate model).

## Discussion

In the current study, we presented the novel hypothesis that hippocampal atrophy may increase susceptibility to symptoms of psychotic disorder following moderate-severe TBI. We based this on literature that: (1) shows hippocampal atrophy in the chronic stages of TBI (atrophy that follows timelines corresponding to the emergence of psychosis),^20^^,^^21^ (2) has implicated the hippocampal head in dopaminergic dysfunction and positive psychotic symptoms,^44^^,^^71^ and (3) revealed that psychosis following TBI is characterized predominantly by positive symptoms.^1^^,^^8^^,^^34^ To explore this possibility empirically, we examined the relationship between hippocampal volume loss and increasing positive psychotic symptoms from 5 to 12 months following injury. Consistent with our hypotheses, worsening scores on the positive symptoms subscale of the PAI were associated with right-sided hippocampal deterioration, both for the whole hippocampus, and for the hippocampal head.

We sought further support for this hypothesis in the form of divergent validity. First, we examined the relationship between worsening positive symptoms of schizophrenia and volume loss in the corpus callosum, for which volume loss has been observed across the same timelines as the hippocampi.^21^ Here, the absence of a significant relationship provided evidence of divergent validity. Second, we examined the relationship between degenerative change in the hippocampi and worsening negative symptom severity. Again, we found no significant relationship. Both relationships also showed small effect sizes. Thus, hippocampal (but not corpus callosum) degeneration was associated with a worsening of the clinical features of schizophrenia that are observed to follow TBI, namely positive (but not negative) symptoms.

### The hippocampal role in psychosis

Observed association between degenerative change of the hippocampal formation and worsening PAI-SCZ-P scores in the present investigation is consistent with evidence from the schizophrenia literature, which has implicated hippocampal dysfunction in both the production of delusions,^72^ and auditory hallucinations,^73^ which are the most common presentations of psychosis after TBI.^2^ These findings are compelling considering that death of certain hippocampal neurons has been found to increase risk of positive psychotic symptoms.^45–47^^,^^71^ This has been reviewed in depth in the context of schizophrenia^44^ and our results are suggestive that this mechanism may bear important implications for post-TBI psychosis as well.

In schizophrenia, this increased risk is attributable to the important hippocampal role in regulating dopaminergic firing and affective salience via control of mesolimbic systems. Briefly, inhibitory, GABAergic neurons of the hippocampus, largely localized to the hippocampal head, exert regulatory control over dopaminergic neurons of the mesolimbic system.^44^^,^^48^^,^^74^^,^^75^ As observed in schizophrenia, degeneration of the hippocampus, and in particular loss of these inhibitory neurons, results in increased excitatory firing downstream; this subsequently produces aberrant elevations in the amplitude of dopaminergic firing to the prefrontal cortex when stimuli are encountered.^45^^,^^46^^,^^76^

**Figure 3:**
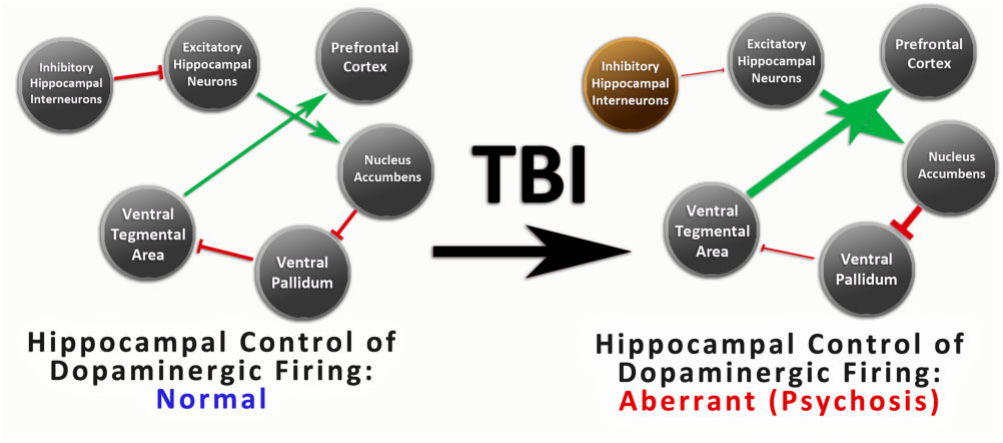
**Outline of novel, neurodegenerative hypothesis.** Through the proposed mechanism, degenerative change in hippocampal neurons (indicated in brown) may result in increased risk of psychotic disorder post-TBI. Loss of inhibitory hippocampal interneurons post-TBI may result in dysregulation of networks controlling amplitude of dopaminergic firing. Differences between a normative network (left) and an altered network (right) are shown. Inhibitory projections are shown in red while excitatory projections are shown in green, with differences in size denoting changes in strength following degenerative change in GABAergic, inhibitory interneurons of the hippocampus. Model of dopaminergic dysregulation pursuant to hippocampal neuronal loss is adapted from relationships described in schizophrenia by Grace 2012. GABA = gamma aminobutyric acid.

In the absence of pathology, hippocampal regulation of this system contributes to the appropriate ascription of relevance, or ‘salience’ to a stimulus, incorporating input of various brain regions to evaluate the relative importance of the stimulus to the organism, in relation to cognitive processes, memories and affective states.^77–79^ However, dysregulated dopaminergic firing in psychosis impairs the ascription of the appropriate salience to encountered stimuli.^31–33^ This neurochemical dysregulation has led to the description of psychosis as a state of ‘aberrant salience’ in which pathological ascription of high salience to internal percepts and external stimuli results in delusions and hallucinations.^31–33^ As such, this represents a compelling mechanism by which hippocampal volumetric decline (and concurrent death/dysfunction of hippocampal neurons) might determine PAI-SCZ-P scores, consistent with the present results (Fig. 3).

Previous work has demonstrated that, post-TBI, significant degeneration occurs within these inhibitory neuronal populations with one study in a murine model of TBI finding GABAergic interneuron loss and consequent reductions in inhibitory hippocampal firing one week post-injury, and a second finding continued decline in inhibitory hippocampal interneurons six months post-injury.^80^^,^^81^ Of relevance, in the subset of TBI survivors who experience post-traumatic temporal lobe epilepsy, the loss of these inhibitory interneurons is understood to be associated with epileptogenesis through a similar mechanism as is proposed here for post-TBI psychosis.^82^ In post-traumatic epilepsy, reduced inhibition from hippocampal interneurons is associated with downstream cortical hyperexcitability evolving along similar timelines as is seen in post-TBI psychosis, with many patients developing seizure disorders years post-injury.^83^ Psychotic symptoms may also present in cases of temporal lobe epilepsy and have been associated with similar hyperactivity of hippocampal neurons projecting to the ventral subiculum-nucleus accumbens-ventral pallidal-VTA pathway, resulting in subsequent overdrive of dopaminergic firing.^84^ As such, analogous loss of hippocampal inhibitory control from the interneurons projecting to these excitatory hippocampal neurons involved in affective salience may be expected to correlate with increased risk of the positive symptoms seen in post-TBI psychosis. Notably, when these GABAergic neuronal populations were experimentally inhibited in a murine model, Nguyen et al.^49^ reported that behavioural changes consistent with psychosis were observed only after the quantity of inhibited neurons reached a certain threshold, highlighting the importance of this neuronal population and perhaps suggesting initial damage alone may be insufficient to increase risk of psychosis through this pathway.^49^ Of particular relevance to TBI, another investigation observed declines in these GABAergic neuronal populations in response to social isolation in the absence of injury.^85^ This suggests the substantial lifestyle alterations that commonly accompany injury may also preferentially impact these key regulatory neurons, perhaps resulting in synergistic contributions of these psychosocial factors to neurodegeneration.

In a subset of patients, progressive degenerative change in the hippocampus (particularly the head) may impact these proposed candidate neurons post-TBI, resulting in disinhibition of downstream, dopaminergic firing, subsequently reinforcing a pathological state of aberrant salience.^44^^,^^46^^,^^71^^,^^76^ When evaluating encountered stimuli, aberrant ascription of affective salience may predispose an individual to experiencing delusions or hallucinations^31–33^ and thus dysfunction of these regulatory systems may present in schizophrenia-like psychosis with greater prevalence of positive symptoms. As such, the proposed involvement of these GABAergic interneurons of the hippocampal head is supported by both the present findings and the reported presentation of post-TBI psychotic disorder.^2^ Future work is required to further verify this proposed mechanism.

### Lateralization of findings

Degenerative change of the right and not left hippocampus was associated with PAI-SCZ-P change scores. This finding is convergent with a growing body of literature that has associated right-sided acquired brain injury with *de novo* psychotic disorder more frequently than left-sided injury.^34^^,^^50–52^^,^^86^^,^^87^ Particularly regarding stroke, right-sided infarction is predominately considered as a precipitator of psychotic disorder (as opposed to left-sided infarction); a recent systematic review found that 86% of patients with post-stroke psychotic disorder had right-sided lesions.^52^ The same review concluded that, comparable to post-TBI psychotic disorder, a substantial delay in onset was common in cases of post-stroke psychosis, implicating contributions of degenerative processes as opposed to the primary lesion and perhaps suggesting a comparable aetiological mechanism. Compellingly, unilateral stroke damages the ipsilateral hippocampus, causing progressive reductions in structural integrity up to years post-infarction.^88^ As such, the right-sided infarctions associated with psychotic symptoms may also cause coincident right-sided hippocampal atrophy as well. Similarly, in Alzheimer’s disease, right-sided hippocampal atrophy has been associated with development of psychotic symptoms.^42^ It may be considered that the convergent findings of the present study may speak to a shared underlying mechanism.

### Limitations and future directions

While the results of this study provide some initial empirical support for the hypothesis that hippocampal volume loss may cause or hasten the onset of psychosis following TBI, there are several important limitations. First, the retrospective study design limited both the outcome measures and the population included in this analysis. Most importantly, the sample contained patients who showed elevations on the PAI-SCZ-P subscale, but none had a clinical diagnosis of schizophrenia. The size of the sample limited the number of variables that could be included in a single model. As well, while the PAI and particularly the PAI-SCZ-P are useful measures of neuropsychiatric functioning with validity for this population,^60^ future researchers may consider employing alternative, gold-standard measures of psychotic symptom severity such as the Positive and Negative Syndrome Scale (PANSS).^89^ The PANSS is sensitive to both positive and negative symptoms of schizophrenia and is a well-established indicator of symptom severity in cases of schizophreniform psychosis, though, unlike the PAI, formal validation of the PANSS has not been performed in the context of TBI.

This investigation was restricted to change scores between the 5- and 12-month time-points; an analysis extending to later time points may identify individuals who develop clinical psychotic disorder beyond the 1–3-year latency period. The significant effect observed with this continuous measure of psychotic symptom severity raises the hypothesis that early degenerative change may be a contributor to risk of psychosis or psychotic prodrome.

Finally, the mechanisms we posit for the relationship between hippocampal degeneration and psychotic symptoms largely concern specific GABAergic, regulatory interneurons. Research is needed that can evaluate degenerative patterns within these specific populations of hippocampal neurons.

### Clinical implications

Validation of the relationships posited here in future research would offer a compelling opportunity for future, clinical intervention research. Rehabilitative therapies targeting hippocampal volume loss may prove capable of mitigating risk of psychosis. For instance, recent investigations of treatments targeting neuroinflammation immediately following TBI have demonstrated efficacy in mitigating loss of these inhibitory hippocampal interneurons following TBI.^90^ In addition, the hippocampus represents a particularly plastic structure and as such, non-invasive, behavioural interventions may bear the potential to offset the degenerative change observed after injury.^91^^,^^92^ In particular, environmental enrichment paradigms (particularly when paired with aerobic exercise) have shown promise in both murine and human models, with the potential to increase hippocampal neurogenesis and functional integration within the hippocampus.^91^^,^^93–97^ Encouragingly, emerging evidence in murine models suggests that environmental enrichment may reduce hyperactivity in the hippocampal head and subsequently reduce dopaminergic hyperresponsivity.^98^ Therefore, interventions integrating this approach may bear particular promise in mitigating risk of post-TBI psychosis, should this proposed mechanism be further validated.

## Conclusion

Post-TBI psychosis is an exceptionally debilitating sequela of injury. However, little is known as to the aetiological processes that underlie altered risk of psychosis in TBI survivors. There is an important role of the hippocampal head in the development of positive, psychotic symptoms outside of the TBI context; in the context of TBI, there are degenerative changes observed in hippocampal head in the chronic stages of injury, not long before the typical onset of post-TBI psychosis. The demonstration of an association between degeneration of the right, hippocampal head and positive (but not negative) psychotic symptoms is compatible with the novel supposition that chronic hippocampal degeneration may play a causal role in the onset of psychosis after moderate-severe TBI. If verified, then treatments that mitigate hippocampal volume loss may thereby mitigate post-TBI psychosis risk.

## Supplementary material

Supplementary material is available at *Brain Communications* online.

## Supplementary Material

fcab026_Supplementary_Data

## Abbreviations

BIC =: Bayesian information criterion
CI =: confidence interval
PAI =: Personality Assessment Inventory
PAI-SCZ-P =: Psychotic Experience Subscale on the Schizophrenia Clinical Scale of the Personality Assessment Inventory
PAI-SCZ-S =: Social Detachment Subscale of the Schizophrenia Clinical Scale of the Personality Assessment Inventory
PTA =: post-traumatic amnesia
TBI =: traumatic brain injury
