# Erratum to: Hippocampal Atrophy is Associated with Psychotic Symptom Severity Following Traumatic Brain Injury

**DOI:** 10.1093/braincomms/fcab122

**Published:** 2021-06-25

**Authors:** Michael J C Bray, Bhanu Sharma, Julia Cottrelle, Matthew E Peters, Mark Bayley, Robin E A Green

Michael J. C. Bray, Bhanu Sharma, Julia Cottrelle, Matthew E. Peters, Mark Bayley, Robin E. A. Green. Hippocampal atrophy is associated with psychotic symptom severity following traumatic brain injury. Brain Communications 2021. doi:10.1093/braincomms/fcab026.

In the originally published version of this manuscript, an author’s surname was spelled incorrectly as Cottrelle’s. The correct full name is Julia Cottrelle. This error has been corrected online.

