# Supplementary material for: Hippocampal atrophy is associated with psychotic symptom severity following traumatic brain injury
Source: Brain Commun. 2021 Mar 9;3(2):fcab026. doi: 10.1093/braincomms/fcab026 (PMC8098106; doi:10.1093/braincomms/fcab026)
Supplement: fcab026_Supplementary_Data [file fcab026_supplementary_data.docx]

**Supplementary Table 1.** Block forced entry of total right HPC (% volumetric change) as a predictor of % change in psychotic symptom severity into a multivariate model containing all CVs.

|  | Estimate | 95% CI | *T*-value | *p*-value | Estimate | 95% CI | *T*-value | *p*-value |
| --- | --- | --- | --- | --- | --- | --- | --- | --- |
| *Block 1: Control Variables* |  |  |  |  |  |  |  |  |
| Age | -0.00422 | (-0.00925, 0.00082) | -1.784 | 0.0946 | -0.00161 | (-0.00636, 0.00314) | -0.727 | 0.4789 |
| Sex | 0.01261 | (-0.15445, 0.17966) | 0.161 | 0.8744 | 0.09270 | (-0.06267, 0.24807) | 1.280 | 0.2215 |
| Marijuana Use | 0.00362 | (-0.00505, 0.01230) | 0.890 | 0.3873 | 0.00757 | (-0.00043, 0.01557) | 2.030 | 0.0618 |
| Years of Education | 0.01200 | (-0.01526, 0.03926) | 0.938 | 0.3630 | 0.01010 | (-0.01305, 0.03324) | 0.936 | 0.3653 |
| Schizophrenia Family History | 0.68020 | (-0.27402, 0.41006) | 0.424 | 0.6777 | 0.11360 | (-0.17842, 0.40563) | 0.834 | 0.4181 |
| Length of Post-Traumatic Amnesia |  |  |  |  |  |  |  |  |
| 1-7 Days | 1.00000 | - | - | - | 1.00000 | - | - | - |
| 1-4 Weeks | 0.04680 | (-0.12955, 0.22315) | 0.566 | 0.5800 | 0.10558 | (-0.05105, 0.26221) | 1.446 | 0.1703 |
| > 4 Weeks | 0.03118 | (-0.12551, 0.18788) | 0.424 | 0.6775 | 0.05288 | (-0.08099, 0.18675) | 0.847 | 0.4111 |
|  |  |  |  |  |  |  |  |  |
| *Block 2: Hippocampal Volume Change* |  |  |  |  |  |  |  |  |
| **% Volume Change, Right Total Hippocampus** |  |  |  |  | **-0.02300** | **(-0.04143, -0.00458)** | **-2.677** | **0.0180** |
|  |  |  |  |  |  |  |  |  |
| *Model Goodness of Fit* | (adj. *R^2^* = -0.05153, *F*_(7, 15)_ = 0.846, *p* = 0.5675) | | | | (adj. *R^2^* = 0.2549, *F*_(8, 14)_ = 1.941, *p* = 0.1328) | | | |
|  |  | | | |  | | | |
| *Comparison of Block 1 and 2 models (ANOVA)* | **Δ*R^2^* = 0.3064, F_(1,14)_ = 7.168, *p* = 0.0180** | | | | | | | |
|  |  | | | | | | | |

**Abbreviations:** ANOVA, Analysis of Variance, 95% CI, 95% Confidence Interval.

**Supplementary Table 2.** Block forced entry of right hippocampal head (% volumetric change) as a predictor of % change in psychotic symptom severity into a multivariate model containing all CVs.

|  | Estimate | 95% CI | *T*-value | *p*-value | Estimate | 95% CI | *T*-value | *p*-value |
| --- | --- | --- | --- | --- | --- | --- | --- | --- |
| *Block 1: Control Variables* |  |  |  |  |  |  |  |  |
| Age | -0.00422 | (-0.00925, 0.00082) | -1.784 | 0.0946 | -0.00223 | (-0.00699, 0.00254) | -1.003 | 0.3328 |
| Sex | 0.01261 | (-0.15445, 0.17966) | 0.161 | 0.8744 | 0.07290 | (-0.08326, 0.22906) | 1.001 | 0.3337 |
| Marijuana Use | 0.00362 | (-0.00505, 0.01230) | 0.890 | 0.3873 | 0.00624 | (-0.00173, 0.01420) | 1.680 | 0.1152 |
| Years of Education | 0.01200 | (-0.01526, 0.03926) | 0.938 | 0.3630 | 0.01196 | (-0.01195, 0.03588) | 1.073 | 0.3014 |
| Schizophrenia Family History | 0.68020 | (-0.27402, 0.41006) | 0.424 | 0.6777 | 0.10443 | (-0.19737, 0.40623) | 0.742 | 0.4703 |
| Length of Post-Traumatic Amnesia |  |  |  |  |  |  |  |  |
| 1-7 Days | 1.00000 | - | - | - | 1.00000 | - | - | - |
| 1-4 Weeks | 0.04680 | (-0.12955, 0.22315) | 0.566 | 0.5800 | 0.07521 | (-0.08157, 0.23198) | 1.029 | 0.3210 |
| > 4 Weeks | 0.03118 | (-0.12551, 0.18788) | 0.424 | 0.6775 | 0.03452 | (-0.10297, 0.17200) | 0.538 | 0.5987 |
|  |  |  |  |  |  |  |  |  |
| *Block 2: Hippocampal Volume Change* |  |  |  |  |  |  |  |  |
| **% Volume Change, Right Hippocampal Head** |  |  |  |  | **-0.02142** | **(-0.04060, -0.00224)** | **-2.396** | **0.0311** |
|  |  |  |  |  |  |  |  |  |
| *Model Goodness of Fit* | (adj. *R^2^* = -0.05153, *F*_(7, 15)_ = 0.846, *p* = 0.5675) | | | | (adj. *R^2^* = 0.20090, *F*_(8, 14)_ = 1.691, *p* = 0.1862) | | | |
|  |  | | | |  | | | |
| *Comparison of Block 1 and 2 models (ANOVA)* | **Δ*R^2^* = 0.2524, *F*_(1,14)_ = 5.739, *p* = 0.0311** | | | | | | | |
|  |  | | | | | | | |

**Abbreviations:** ANOVA, Analysis of Variance, 95% CI, 95% Confidence Interval.

**Supplementary Table 3.** Stepwise selection method for social withdrawal modelling.

|  | **Candidate parameter** | **SS** | **RSS** | **BIC** |
| --- | --- | --- | --- | --- |
| **Step 1** | ***No further addition*** | - | 1.178 | -65.188 |
|  | Right HPC head % volume change, 5-12 months | 0.087 | 1.090 | -63.781 |
|  | Pre-injury marijuana use | 0.061 | 1.117 | -63.224 |
|  | Sex | 0.040 | 1.137 | -62.813 |
|  | Age | 0.022 | 1.155 | -62.452 |
|  | Education | 0.006 | 1.171 | -62.122 |
|  | Family history of Schizophrenia | 0.001 | 1.176 | -62.031 |
|  | PTA | 0.013 | 1.165 | -59.078 |

**Abbreviations:** BIC, Bayesian Information Criterion; HPC, Hippocampus; PTA, Post-Traumatic Amnesia; SS, Sum of Squares; RSS, Residual Sum of Squares
